# The effects of soft tissue lateral release on the stability of the ligament complex of the knee

**DOI:** 10.1007/s00402-020-03422-6

**Published:** 2020-03-30

**Authors:** Florian Völlner, Florian Herl, Felix Greimel, Achim Benditz, Tobias Renkawitz, Joachim Grifka, Benjamin Craiovan, Markus Weber

**Affiliations:** Department of Orthopaedic Surgery, Regensburg University Medical Centre, Asklepios Klinikum Bad Abbach, Kaiser-Karl-V-Allee 3, 93077 Bad Abbach, Germany

**Keywords:** Lateral release, Knee balancer, Ligament balance, Ligament stiffness, Soft-tissue balance, Stability, Total knee arthroplasty

## Abstract

**Purpose:**

Valgus deformity presents a particular challenge in total knee arthroplasty. This condition regularly leads to contractures of the lateral capsular ligament complex and to overstretching of the medial ligamentous complex. Reconstruction of the knee joint kinematics and anatomy often requires lateral release. However, data on how such release weakens the stability of the knee are missing in the literature. This study investigated the effects of sequential lateral release on the collateral stability of the ligament complex of the knee in vitro.

**Methods:**

Ten knee prostheses were implanted in 10 healthy cadaveric knee joints using a navigation device. Soft tissue lateral release consisted of five release steps, and stiffness and stability were determined at 0, 30, 60 and 90° flexion after each step.

**Results:**

Soft tissue lateral release increasingly weakened the ligament complex of the lateral compartment. Because of the large muscular parts, the release of the iliotibial band and the M. popliteus had little effect on the stability of the lateral and medial compartment, but release of the lateral ligament significantly decreased the stability in the lateral compartment over the entire range of motion. Stability in the medial compartment was hardly affected. Conversely, further release of the posterolateral capsule and the posterior cruciate ligament led to the loss of stability in the lateral compartment only in deep flexion, whereas stability decreased significantly in the medial compartment.

**Conclusion:**

Our study shows for the first time the association between sequential lateral release and stability of the ligamentous complex of the knee. To maintain the stability, knee surgeons should avoid releasing the entire lateral collateral ligament, which would significantly decrease stability in the lateral compartment.

## Introduction

Valgus deformity, which affects about 10% of patients undergoing total knee arthroplasty (TKA), presents a major challenge to surgeons. Valgus deformity is characterised by bony malformations, such as lateral cartilage erosion, lateral condylar hypoplasia and metaphyseal femur as well as tibial plateau remodelling. A further characteristic of valgus deformity is asymmetrical ligament proportion, such as elongation of the medial parts and contractions of the lateral parts [iliotibial band (ITB), lateral collateral ligament (LCL), popliteus muscle (POP), posterolateral capsule (PLC) and hamstring muscles (HAR)] of the knee ligament complex [10, 15]. As long as the medial ligament complex is functionally intact and the lateral ligament complex is not too tight, valgus deformity can be corrected by lateral release of the capsular ligament complex.

Various correction techniques have been described in the literature. Some authors achieved good clinical results with sequential lateral release [2, 10, 12, 15, 17, 24], whereas Krackow et al. preferred detachment of the ITB and LCL followed by detachment of the POP and PLC [10]. Ranawat et al. first released the PCL and then the PLC followed by pie crusting of the ITB and LCL [15]. Matsueda et al. first released the ITB followed by release of the POP, LCL, PLC and PCL [12]. In contrast, Böttner et al. used a standardised soft tissue release technique of the ITB, PLC, LCL and the anterior lateral ligament, which had shown excellent clinical results at the 2-year follow-up [2]. According to Whiteside et al., who released the LCL and POP in flexion contracture and the ITB and PLC in extension contracture, release depends on the functional effect [24].

Despite these study results, the sequence of lateral soft tissue release to achieve the best alignment without causing instability of the knee is still under debate in the literature. The hypothesis of the current study was that lateral release weakens the knee ligament complex and that over-release may cause secondary instability. Therefore, sequential lateral release was conducted as a part of total knee arthroplasty, and stiffness and stability of the knee ligament complex were determined at 0, 30, 60 and 90° flexion. The study clearly showed the stage at which instability occurs.

## Materials and methods

### Cadaver specifications

In the current study, unhurt full body specimens embalmed by Thiel’s method were used. Stability was examined in five left and five right knee joints. None of the knees showed any deformity, and none of the specimens had previously undergone surgery on the lower extremities. At the time of examination, the knee joints were clinically stable. All knees and hip joints had a full range of motion. Mean leg axis was 174.8 ± 3.4°, the anatomical lateral distal femur angle (aLDFA) 80.7 ± 2.4°, the anatomical medial proximal tibia angle (aMPTA) 86.2 ± 2.1° and the joint line convergence angle (JLCA) 1.0 ± 0.8°.

### Surgical technique and in vitro measurements

Stiffness was determined during the standard surgical routine for total knee arthroplasty established at our clinic [21–23]. After mid-line skin incision, the capsule was opened using the medial parapatellar approach. The anterior cruciate ligament and the menisci were resected. Two Schanz screws were bicortically drilled into the femur and the tibial plateau outside the joint capsule to avoid soft tissue damage. Subsequently, the passive optical reference arrays were fixed (Brainlab AG, Munich, Germany). After registration of the knee joint, an 8 mm bone/cartilage of the healthy lateral compartment of the tibia was resected with a 4° slope in the frontal plane perpendicular to the mechanical axis of the tibia. In the next step, the femoral trial component was implanted with 3° external rotation to achieve the best coverage of the femoral bone (DePuy PFC Sigma cruciate retaining, Depuy, Warsaw, IN). The ligament complex was thereby protected by retractors.

**Fig. 1:**
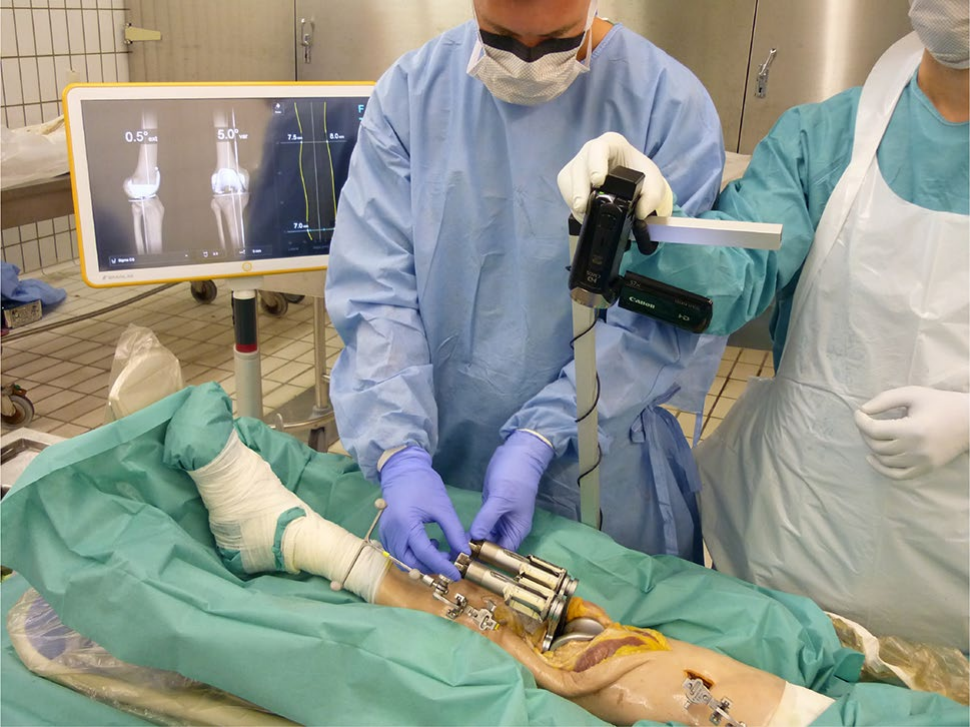
Setup of the experiment with a CPM machine, navigation system, knee balancer in situ and camera

To determine stiffness, a knee balancer was placed into the extension gap between the tibia and the femoral trail component. The leg was straightened, and a preload of about 10–20 N was applied to the medial and lateral ligamentous complex. The load was then slowly spread to 180 N. The expansion was recorded on video, and the tapes were evaluated after surgery. The procedure was repeated at 30, 60 and 90° flexion. Knee flexion was adjusted with a continuous passive motion device and monitored by the navigation system (Fig. 1). After the knee joint was examined over the full range of motion, sequential release was conducted stepwise, and measurements were carried out as described below. All measurements were repeated twice. Arthroplasty was conducted by one surgeon with 10 years of experience.

### Sequential lateral soft tissue release

The sequential lateral release technique used had been modified according to the recommendations by Matsueda et al. [12]. The structures were either released subperiosteally or by means of the subligamentous technique in five successive steps using a scalpel or a Cobb elevator:

1. Release of the iliotibial band at the level of the joint line,
2. Release of the femoral attachment of the popliteus muscle,
3. Release of the lateral collateral ligament from the femoral condyle,
4. Release of the posterolateral capsule from the femoral insertion, and
5. Release of the entire posterior cruciate ligament on the tibial side.

### Reconstruction of the force–elongation curve

The force–elongation curve was reconstructed with the knee balancer (P.F.C Sigma and LCS Complete EGF Instrumentation of Depuy Synthes, Warsaw, USA), which displays the applied force and elongation at all times. Therefore, the device had been validated prior to the study [23].

To calculate stiffness, the videos were read into Matlab (Mathworks, Natick, USA), and every 10th frame was extracted. The applied force and the elongation of the capsule ligaments were calculated, and the force–elongation curve was plotted. The slope of the linear section (from approx. 80 N) of the force–elongation curve was defined as the stiffness of the ligament complex and represents the structural properties. Slope, box plots and 3D plots were calculated with Excel software (Microsoft Corp. Redmond, USA).

To calculate the stability, the mean value of stiffness was set to 100% at the native knee joint. Decreased stability as a result of the release refers to the respective native value at 0, 30, 60 and 90° flexion.

### Statistical analysis

For statistical analysis, measurements are presented as box plots. Group comparisons were done with the Mann–Whitney *U* test because of the non-normal distribution of the data. The primary hypothesis was tested at a two-sided 5% significance level. IBM SPSS Statistics 23 (SPSS Inc, Chicago, IL, USA) was used for analysis.

## Results

**Fig. 2:**
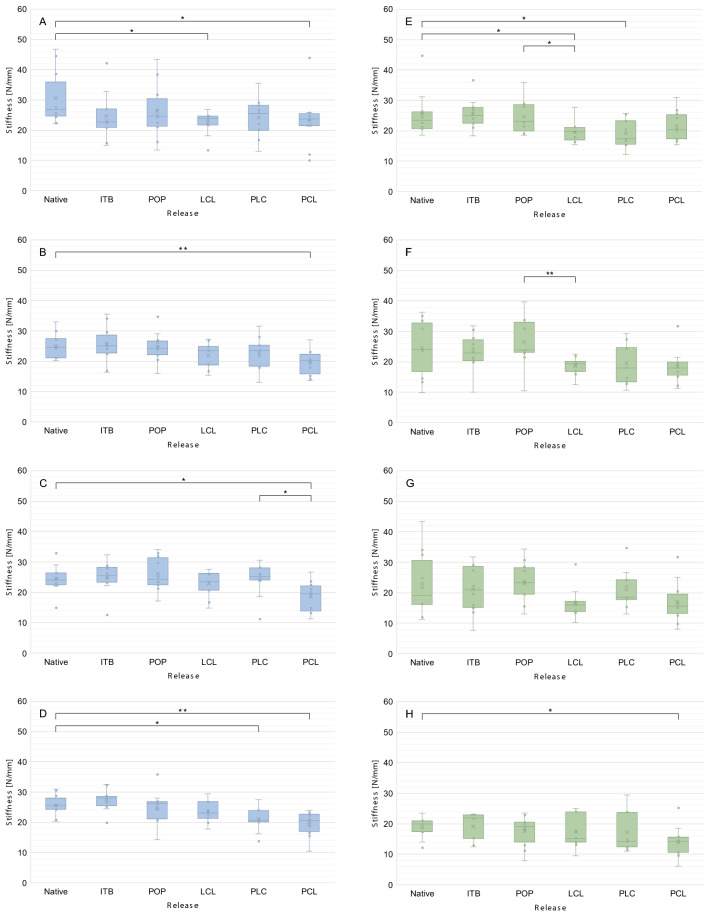
Stiffness of the medial (blue, **a** full extension, **b** 30° flexion, **c** 60° flexion, **d** 90 degrees flexion) and lateral (green, **e** Full extension, **f** 30° flexion, **g** 60° flexion, **h** 90° flexion) compartment depending on the release of the lateral knee ligament complex. In the medial compartment, stiffness decreases with increasing release, whereas stiffness at the lateral compartment remains the same. *p < 0.05, **p < 0.01

**Fig. 3.**
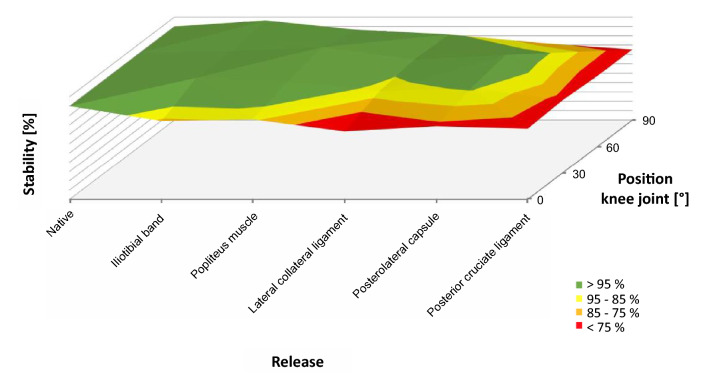
3D plot of the stability of the medial ligament complex depending on the lateral release and flexion of the knee joint. A decrease in the stability as a result of the release refers to the respective native value (set as 100%) at 0°, 30°, 60° and 90°

**Fig. 4.**
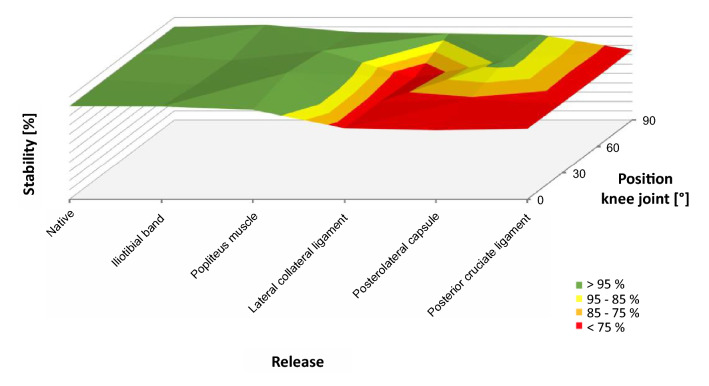
3D plot of the stability of the lateral knee ligament complex depending on the lateral release and flexion of the knee joint

Tables 1 and 2 show the summary of median values for stiffness and the interquartile range depending on knee joint flexion and release steps. Figure 2 shows stiffness as a function of release for the medial (A–D) and lateral (E–H) compartments. Figure 3 shows the 3D plots of stability for the medial compartment and Fig. 4 for the lateral compartment.

**Table 1:** Summary of mean stiffness [N/mm] and the interquartile range [N/mm] for the medial compartment depending on release steps and knee joint flexion

Flexion knee joint	0°	30°	60°	90°
Native	26.8 (11.2)	24.5 (6.3)	24.0 (3.6)	25.6 (3.7)
Iliotibial band	22.6 (4.6)	25.1 (6.0)	26.2 (5.0)	28.0 (2.2)
Popliteus muscle	24.6 (9.1)	23.9 (4.2)	24.2 (8.7)	25.1 (5.7)
Lateral collateral ligament	23.9 (2.0)	23.5 (6.1)	23.5 (5.6)	23.1 (5.5)
Posterolateral capsule	25.5 (8.2)	23.6 (6.9)	25.6 (3.8)	22.4 (3.7)
Posterior cruciate ligament	23.8 (2.6)	20.1 (6.6)	19.5 (8.2)	20.5 (5.7)

**Table 2:** Summary of mean stiffness [N/mm] and the interquartile range [N/mm] for the lateral compartment depending on release steps and knee joint flexion

Flexion knee joint	0°	30°	60°	90°
Native	22.7 (5.1)	24.1 (16.0)	19.2 (14.3)	20.1 (3.4)
Iliotibial band	24.0 (4.6)	22.9 (6.9)	20.9 (13.4)	21.8 (7.6)
Popliteus muscle	23.3 (8.8)	24.0 (10.0)	23.2 (8.7)	19.2 (6.5)
Lateral collateral ligament	19.7 (4.3)	19.4 (3.3)	15.0 (3.1)	15.3 (10.0)
Posterolateral capsule	17.3 (7.7)	18.1 (11.2)	18.4 (5.6)	14.2 (11.4)
Posterior cruciate ligament	20.3 (8.0)	17.1 (4.5)	15.3 (4.1)	13.8 (5.1)

### Medial compartment

Release of the ITB and POP did also neither significantly change stiffness nor stability of the knee joint at the medial compartment. Release of the LCL only significantly decreased stiffness in full extension (from 26.8 to 23.9 N/mm; *p* = 0.022) but not in flexion (Fig. 2; Table 1). This finding corresponded to a loss of stability of almost 10–25% (Fig. 3). Release of the PLC had no influence on the stiffness and stability of the medial compartment. Only release of the PCL led to significant weakening in flexion (30° *p* = 0.010, 60 degrees: *p* = 0.019, 90° *p* = 0.002), which corresponded to a loss of stability of up to 25% over the entire range of motion.

### Lateral compartment

In the lateral femorotibial compartment, release of the ITB and POP did also not affect the stiffness and stability of the knee joint. Release of the LCL at the femoral insertion led to significant weakening from 22.7 to 19.7 N/mm (*p* = 0.022) in extension from 24.0 to 19.4 N/mm (*p* = 0.102), in 30° flexion, from 19.2 to 15.0 N/mm (*p* = 0.191) in 60° flexion and from 20.1 to 15.3 N/mm (*p* = 0.568) in 90° flexion (Fig. 2; Table 2). These results corresponded to a loss of stability of 20–30% of the initial value (Fig. 4). Release of the PLC slightly decreased stiffness, whereas release of the PCL only significantly decreased stiffness from 20.1 to 13.8 N/mm in 90° flexion (*p* = 0.028).

## Discussion

The present study describes the relation between sequential lateral release and the stiffness and stability of the medial and lateral knee ligament complex in vitro. The most important finding of this study was that the LCL is the main passive stabiliser in the lateral compartment in extension and in light flexion up to 60°. From 60° up, stabilisation of the knee joint seems to be increasingly carried out by the PCL. Furthermore, the PCL is the main stabiliser in the medial compartment over the entire range of motion.

In the current study, stiffness and stability were determined by spreading the medial and lateral compartment to determine the mechanical properties of the entire knee joint. Except for a study by Völlner et al. which describes the influence of sequential medial release on the capsular ligament complex there are only studies examining separate ligaments available in the literature [13, 16, 19, 29], a direct cross-comparison with the present results is not possible. The results of this study must, therefore; be discussed primarily on the basis of anatomical considerations.

As mentioned above, the method of gradual and reproducible sequential lateral release used in this study had been modified according to the suggestions of Matsueda et al. [12]. Therefore, the ITB and POP were released at the level of the joint line respectively at femoral insertion, which neither decreased stiffness nor the stability of the medial and lateral compartments (see Tables 1, 2 and Figs. 2, 3, 4). Anatomically, both structures are characterised by a large muscular component [6, 7]. The iliotibial tract originates from the tendinous fibres of the tensor fasciae latae muscle, the gluteus maximus muscle and the fascia of the gluteus medius muscle and extends from the anterior superior iliac spine via the hip and knee to the condylus lateralis tibiae to the condyle termed Gerdy's tubercle. The popliteus muscle originates at the lateral condyle of the distal femur and attaches to the articular capsule of the knee joint and the posterior surface of the tibia [18]. Because muscles are naturally relaxed in cadavers, neither the ITB nor the POP can provide any stability, also in the native state. The current results showed that both structures are primarily dynamic stabilisers in vivo because of their muscular components. The influence of these structures on the stability of the knee joint could not be measured in the present experimental setup, so that only passive stabilisation due to ligaments and capsule structures was recorded. In valgus knee joints, both the ITB and the POP are contracted as malalignment progresses. To reconstruct the leg axis, release of both structures is necessary in many patients. Because neither the ITB nor the POP contribute to passive stabilisation in physiological leg axes, both structures can be generously released without the risk of primary instability.

In the next step, the LCL was released. This ligament extends from the head of the fibula to the lateral femoral condyle and is maximally stretched in full extension of the knee joint [4, 14, 25]. The attachment of the ligament to the femur is flat and located in dorsal direction to the centre of rotation. Flexion of the knee joint therefore leads to an approximation of the two insertions and enables the internal and external rotation of the tibia. Because of the almost always orthogonal course of the ligament to the joint surface—in contrast to other passive stabilisers in extension—the LCL is the ideal passive stabiliser of the knee joint for varus stress from a biomechanical point of view. This fact was confirmed by the current measurements. The release of the LCL significantly decreased stiffness in both the lateral and medial compartments in full extension, corresponding to a loss of stability of approx. 25% (Fig. 4). Furthermore, a highly significant relative decrease in stiffness and stability in the lateral compartment was observed at 30 and 60° flexion, but no loss of stiffness and stability at 90° flexion (Figs. 2, 4). The LCL is therefore to be regarded as the main stabiliser at 0–60° flexion and should therefore not be completely released to prevent instability in extension and slight flexion.

In the next step, the PLC was released, which consists of a large number of different ligament structures. The most important structures are the popliteus tendon and the popliteofibular ligament. Because of their transverse course to the leg axis, these structures primarily resist next to varus angulation particularly external rotation and posterior translation of the tibia [5, 18]. Therefore, it is not surprising that the release of the PLC has no influence on the stability on the knee joint in axial direction. Release of these structures primarily leads to rotational and translation instability due to the course of the ligaments.

The last step included release of the posterior cruciate ligament. The PCL is the strongest ligament of the knee and consists of multiple bundles [5]. This ligament is the primary restraint to posterior tibial translation and a secondary restraint to internal tibial rotation [5, 9]. The roll and slide mechanism of the knee joint in flexion changes the direction of the posterior cruciate ligament. On the one hand, the fibres straighten up during flexion to the tibia axis and, on the other hand, more fibres are recruited [5]. From an anatomical point of view, the PCL is an ideal stabiliser in flexion for the load case used in this study. This fact was also reflected in the measurements, which showed a significant drop in stiffness and stability in 90° flexion compared to the native joint. The PCL is therefore to be seen as the main stabiliser in the lateral compartment in increased flexion and seems to be the functional counterpart to the LCL. In the medial compartment, a significant drop was observed across the entire ROM.

The current measurements showed values for median stiffness in full extension of 26.8 N/mm (IQR 11.2 N/mm) for the medial ligament complex and 23.5 N/mm (IQR 5.5 N/mm) for the lateral ligament complex. In contrast, literature reports have only described stiffness measurements for isolated ligaments so far [16, 19, 26, 28]. The study by Sugita et al., for instance, yielded a linear stiffness value of 58.1 ± 22.8 N/mm for the lateral collateral ligament in 10 cadaveric knees. A similar result was reported by Wilson et al. [28], who measured stiffness values of 59 ± 12 N/mm in a tensile test for the lateral collateral ligament and of 63 ± 14 N/mm for the medial collateral ligament. These values were higher than the present measurements, probably for various complex reasons. In contrast to the examination of only separate ligaments in other studies, the current study determined stiffness of the entire knee joint. Therefore, the direction of tensile force could not be aligned with the ligament structure itself but was only examined at different joint positions. The fibres of the individual ligaments always run transversely to the direction of the tensile force, which reduces stiffness values. Another reason is that only ligaments but not the bony attachment of the ligament have been examined in most studies so far. However, elongation may also be present at the bony attachment, which would result in lower stiffness values. Another reason for the difference in findings could be the use of Thiel-fixed whole-body preparations in the current study, which are characterised by their lifelike histological structure, colour and ligament consistency [20]. Nevertheless, fixation may result in falsified biomechanical properties [3]. Wilke et al. showed the biomechanical comparability of the nonlinear load–deformation characteristic of spinal motion segments in comparison to tests with fresh frozen cadavers, but with increased values for flexibility [27]. In a separate study on the ligamentous properties of the knee joints, Völlner et al. did not find any significant differences to intraoperative comparative measurements in vivo [21].

This study has several limitations. The first limitation is the low number of 10 knee joints which just corresponds to the minimum quantity for evaluating any new method established by Audigé et al. [1]. A further limitation is the use of knee joints without any deformity, which is also similar to other studies [8, 11, 24]. However, the effect of the described release sequence may differ from the clinical situation. Contractions of the lateral capsular ligament complex are usually a characteristic of valgus knee joints, whereas the medial parts are elongated. Contractures may result in different degrees of stiffness of the ligaments and have to be released to correct misalignments of the axis. However, in the corrected position, the knee joint must be guided by the ligaments. The conclusion that the lateral ligament should not be completely released is thus correct. Because of the insufficient number of defined valgus knee joints in cadaveric specimens available, the authors of the current study plan to investigate the effect of sequential lateral release in knees with valgus deformity in vivo to be able to make further statements about the selective release of the capsular ligament complex. Furthermore, in the current study, only passive stabilisers such as ligaments and capsule portions were recorded, but no active stabilisation through the knee joint musculature could be found, which is probably rather high in vivo. Furthermore, the present study only represents a snapshot because stiffness and stability of the knee joint may also be affected by the healing process and by scarring after total knee arthroplasty. In this study, axial load was applied by means of a ligament balancer; thus, changes in rigidity and stability could only be calculated in one tensile direction, which did not allow any conclusion about instability due to rotation or complex loading. However, the present model mirrors the most common load case.

## Conclusion

This is the first study that shows the association between sequential lateral release and weakening of the knee ligament complex. The LCL is the main stabiliser in the lateral compartment in extension and in flexion up to 60° and should thus not be released under any circumstances. The PCL seems to be the functional counterpart to the LCL in the lateral compartment stabilising the knee joint at increasing flexion. In the medial compartment, the PCL has stabilising properties over the entire ROM. Release of ITB, POP and PLC has no effect on the stiffness and axial stability of the knee joint.

## Abbreviations

HAR: Hamstrings muscle
ITB: Iliotibial band
LCL: Lateral collateral ligament
MCL: Medial collateral ligament
PCL: Posterior cruciate ligament
PLC: Posterolateral capsule
POP: Popliteus muscle
TKA: Total knee arthroplasty
